# Waist circumference and risk of 23 site-specific cancers: a population-based cohort study of Korean adults

**DOI:** 10.1038/s41416-018-0214-7

**Published:** 2018-10-17

**Authors:** Kyu Rae Lee, Mi Hae Seo, Kyung Do Han, Jinhyung Jung, In Cheol Hwang, on behalf of the Taskforce Team of the Obesity Fact Sheet of the Korean Society for the Study of Obesity

**Affiliations:** 1 Department of Family Medicine, Gachon University Dong Incheon Gil Hospital, Incheon, South Korea; 2 Division of Endocrinology and Metabolism, Department of Internal Medicine, Gumi Sonnchunhyang Hospital, Gumi, South Korea; 3 Department of Biostatistics, Catholic University College of Medicine, Seoul, South Korea; 4 Department of Family Medicine, Gil Medical Center, Gachon University College of Medicine, Incheon, Republic of Korea

**Keywords:** Cancer epidemiology, Risk factors

## Abstract

**Background:**

Large waist circumference (WC) is a risk factor for several site-specific cancers, but a large-scale systematic investigation across all common cancers adjusted for potential confounders has not been conducted. This study aimed to evaluate the possible links between WC and common cancers.

**Methods:**

We prospectively examined the association between WC and the risk of cancers in a 7-year cohort study of nearly 22.9 million Korean adults. Using the claims database merged with the national health check-up data, we fitted proportional hazard models to investigate associations between WC and 23 of the most common cancers, with adjustment for potential confounders, including body mass index (BMI). We also evaluated the modification of BMI on the relationships between WC and the incidence of cancer.

**Results:**

A total of 769,871 cancer cases were identified. WC was positively associated with 18 of 23 cancers, and the effects varied substantially by site in each sex. The modification of BMI on the WC-cancer association also varied across the cancer site; in most cases it mitigated the association. For cancers of the oral cavity, larynx, oesophagus, lung, and premenopausal breast, the BMI adjustment reversed the association toward being positive (all *P*_trend_ < 0.001).

**Conclusions:**

Central obesity, independent of general obesity, was associated with the risk of several cancers. The heterogeneity in the mediating effects of BMI suggests that different mechanisms are associated with different cancer sites. Based upon these findings, active strategies to monitor and prevent central obesity should be implemented.

## Introduction

Obesity is an established risk factor for mortality and various chronic diseases. A pooled analysis of 57 prospective studies has documented that each 5 kg/m^2^ higher body mass index (BMI) was associated with a 29% increase in mortality^1^. Furthermore, results from the EPIC (European Prospective Investigation into Cancer and Nutrition) emphasised the additive use of the central obesity index on predicting the risk of death^2^. Given the inexorable rise in obesity worldwide in recent years^3^, understanding the effects of obesity on major health outcomes is urgent and has not been sufficiently emphasised.

There is increasing evidence that obesity increases the risk of certain types of cancers^4,5^. However, most researchers have emphasised the role of overall obesity (generally assessed as BMI), rather than central obesity, in the etiologies of these malignancies^6^. Because metabolic derangement supported by insulin and insulin-like growth factor (IGF) is a possible mechanism in carcinogenesis^7^, central obesity may present a higher threat to the risk of cancer than to general obesity. Moreover, with regard to tumour angiogenesis and cell proliferation, intra-abdominal fat has been hypothesised to be biologically different from fat in other areas^8,9^.

Comprehensive reviews have recently suggested that central obesity increases the cancer risk in various sites, including the gastrointestinal tract^10–12^, biliary tract^13^, lung^14^, breast^15^, thyroid^16^, head and neck^17^, and genital tract^18–20^. However, there are important limitations to these studies. Individual studies have often had insufficient power, and potential confounders have been inconsistent across studies. In addition, many studies have used self-reported WC data, which probably underestimated the true WC, and there have been few reports of the effects of BMI adjustment on the associations between WC and cancer risk^21,22^. Furthermore, cumulative meta-analyses of observational studies have inherent limitations, including publication biases and heterogeneity^23,24^.

Our aim was therefore to investigate the possible associations between WC and the most common site-specific cancers in a single population using a large-scale nationwide claims database. To better understand the role of abdominal obesity on cancer development, we additionally examined the effects of BMI adjustment on the WC-cancer association.

## Methods

### Design and participants

We used the Korean nationwide claims database and biennial medical examination data provided by the Korean National Health Insurance Corporation (NHIC). In the Republic of Korea, comprehensive medical care of nearly all Koreans (97%) is covered by single insurance from the NHIC. The information contained the enrollees’ demographics, utilisation of medical facilities, disease code registered by clinicians, and pharmacy dispensing claims. Data from medical examinations included health-related surveys using standardised questionnaires, height, weight, blood pressure, and fasting laboratory findings, such as serum glucose and total cholesterol. The WC measurements were added in 2009. Using the survey dataset, we extracted information about the medical history and health-related habits such as smoking, alcohol consumption, and physical activity. The questionnaires, which were reviewed by a trained staff, were self-reported.

We used the data of a 7-year cohort (2009–2015) provided by the NHIC for research purposes. We identified 23,452,862 adults ≥ 20 years of age who received the national health examination service at least once from 2009 to 2012. Individuals who had been diagnosed with any cancer (*n* = 448,468, 1.9%) before 31 December 2008 or who had any missing data on baseline characteristics (*n* = 125,189, 0.5%) were excluded. Finally, 22,879,205 adults were followed-up to the date of any cancer diagnosis, or death or 31 December 2015, whichever was first. For uterine or ovarian cancer, we censored women undergoing hysterectomy or oophorectomy due to benign causes at that time point, respectively. The follow up was a mean of 5.3 ± 1.2 years ( ± standard deviation) after the WC measurements. During the study follow up, 769,871 cancers occurred (385,200 among males; 384,671 among females): 10,981 in oral cavity, 8746 in oesophagus, 132,593 in stomach, 149,397 in colorectum, 61,673 in liver, 20,172 in biliary tract, 45,927 in pancreas, 4926 in larynx, 72,133 in lung, 2963 malignant melanoma, 74,835 in breast, 15,842 in cervix, 9726 in uterus, 13,155 in ovaries, 52,492 in prostate, 1505 in testis, 17,204 in kidney, 20,251 in bladder, 10,617 in brain, 156,315 in thyroid, 15,325 lymphoma, 5216 multiple myeloma, and 8576 leukaemia. Previous studies have already used the NHIC database^25,26^.

### Data collection and processing

The primary outcome was newly developed cancer. The diagnosis of cancer was ascertained by the Serious Disease Registry, a nationwide registry identifying persons who needed greater medical expense benefits because of serious or rare diseases, including malignant neoplasm^25,27^. Within the program, the NHIC had sent specific diagnostic criteria to physicians for copayment reductions, and health institutions were required to review the physicians’ diagnoses. Therefore, misclassification is negligible, and the cancer diagnosis is considered valid. Cancer cases were classified as follows according to the International Classification of Disease for Oncology-10^th^ edition (ICD-10): oral cavity (C00–14), oesophagus (C15), stomach (C16), colorectum (C18–21), liver (C22), biliary tract (C23–24), pancreas (C25), larynx (C32), lung (C33–34), malignant melanoma (C43), breast (C50), cervix (C53), uterus (C54–55), ovaries (C56), prostate (C61), testis (C62), kidney (C64), bladder (C67), brain (C70-72), thyroid (C73), lymphoma (C82–86), multiple myeloma (C90), and leukaemia (C91–95).

BMI and WC were used as an index of overall and central adiposity, respectively. The data on anthropometry were collected by direct measurements at medical institutions equipped with facilities and staff approved by the regulations defined by the KNHIC^28^. The categories of WC were based on quintiles in our cohort. BMI was calculated as weight in kilograms divided by the square of height in meters (kg/m^2^). Individuals were categorised into four groups according to BMI following the World Health Organization recommendations for Asians as follows: underweight, <18.5; normal, 18.5–22.9; overweight, 23.0–24.9; and obese, ≥25.0^29^.

Menopausal status was set at the age of 50 years, representing the usual menopausal age of Korean women^30^. Socioeconomic status, estimated by the average insurance premium per month, was classified into quartiles. Smoking status was categorised into three groups as a non-, former, or current smoker. Alcohol consumption was categorised into three groups as a non-, moderate (<30 g per day), or heavy drinker (≥30 g per day). Regular physical activity was defined as exercise ≥one session per week. A comorbid condition was defined primarily based on the combination of past history and the use of ≥1 drugs for the corresponding disease, which included hypertension (ICD-10 code, I10–13/15), type 2 diabetes (E11–14), and hyperlipidemia (E78). Individuals who had abnormal findings in the health examination were considered as patients with the corresponding parameters: ≥140/90 mmHg of blood pressure, 126 mg/dL of fasting plasma glucose, and 240 mg/dL of total cholesterol.

### Statistical analysis

Analyses were performed separately by sex, as appropriate. Cox proportional hazards models, with attained age as the underlying time metric, estimated the hazard ratio (HR) and 99% confidence interval (CI) for the associations of WC with cancer risk, considering potential confounders such as age (continuous), three health-related habits, three co-morbidities, and the BMI (continuous). In the Cox model, smoking status and alcohol use were included as 3 categories, respectively. The follow-up period began on the date of anthropometric assessment. In addition, the WC results were presented with and without adjustment for the BMI. All Cox models were tested for and met the proportional hazards assumption. SAS software, version 9.4 (SAS Institute, Cary, NC, USA) was used for all statistical analyses. A two-sided *p*-value < 0.05 was considered statistically significant.

## Results

Table 1 lists the baseline characteristics of participants across the WC quintiles by sex. Centrally obese individuals were more likely to be elderly and to have a high BMI and increased comorbidites. Current smoking was inversely associated with WC categories, and heavy drinkers were more common in centrally obese males and centrally lean females.

**Table 1:** Baseline characteristics by waist circumference quintile (cm) in the Korean National Health Insurance Cohort, 2009–2015

%	Male					Female				
	Q1 (≤76)	Q2 (77–81)	Q3 (82–85)	Q4 (86–90)	Q5 (≥91)	Q1 (≤68)	Q2 (69–73)	Q3 (74–77)	Q4 (78–83)	Q5 (≥84)
No.	2,160,039	2,536,624	2,294,267	2,511,520	2,148,838	2,309,344	2,330,322	1,908,544	2,354,680	2,325,027
Follow up, years	5.4 ± 1.2	5.4 ± 1.2	5.4 ± 1.2	5.4 ± 1.2	5.3 ± 1.2	5.2 ± 1.2	5.3 ± 1.2	5.3 ± 1.2	5.3 ± 1.2	5.3 ± 1.3
Age, year										
20–39	49.4	39.0	31.2	27.4	27.7	52.9	29.5	17.7	11.0	8.2
40–59	35.8	44.9	50.0	50.9	48.1	40.8	57.7	60.4	55.5	45.1
60–79	13.6	15.4	18.3	20.8	23.2	5.5	11.8	20.5	31.5	43.6
≥80	1.2	0.9	0.8	0.9	1.0	0.8	1.0	1.4	2.1	3.1
Mean ± SD	42.1 ± 14.8	44.8 ± 13.8	47.0 ± 13.4	48.3 ± 13.3	48.8 ± 13.7	38.5 ± 12.7	45.0 ± 13.0	49.6 ± 13.0	53.7 ± 13.0	57.2 ± 13.3
Body mass index, kg/m^2^										
<18.5	11.4	0.8	0.2	0.1	0.03	23.7	3.3	1.0	0.4	0.1
18.5–22.9	76.9	54.4	26.6	9.2	1.5	73.2	76.0	51.0	25.7	5.8
23.0–24.9	10.0	34.2	44.1	32.5	9.5	2.7	17.5	34.4	37.7	17.2
≥25.0	1.6	10.6	29.1	58.3	89.0	0.4	3.2	13.7	36.2	76.8
Mean ± SD	20.8 ± 1.9	22.8 ± 1.8	24.1 ± 1.8	25.4 ± 1.9	27.9 ± 2.6	19.7 ± 1.7	21.6 ± 1.8	23.0 ± 2.0	24.4 ± 2.2	27.2 ± 3.1
Current smoking	50.9	46.1	43.4	42.1	42.3	4.9	4.2	3.8	3.6	3.9
Heavy drinking	11.0	13.0	14.0	15.3	17.4	1.4	1.3	1.2	1.1	1.1
Regular physical activity	17.9	20.2	20.7	20.4	19.2	12.3	15.7	16.9	16.9	15.3
Hypertension	13.3	20.2	26.9	33.9	45.3	6.2	13.1	21.5	32.1	48.6
Type 2 diabetes	4.8	7.5	10.2	12.9	17.9	1.5	3.3	5.9	9.8	17.8
Hyperlipidemia	7.6	13.4	18.2	22.4	27.9	6.7	13.0	19.8	27.1	36.0
Menopause	—	—	—	—	—	18.3	34.9	50.1	63.1	73.2
Socioeconomic status, low^a^	20.2	17.4	16.8	16.8	17.7	23.6	24.9	25.2	25.0	25.1

*SD* standard deviation.

All *P*_trend_ < 0.001.

^a^Lowest quartile of the insurance premium

**Fig. 1:**
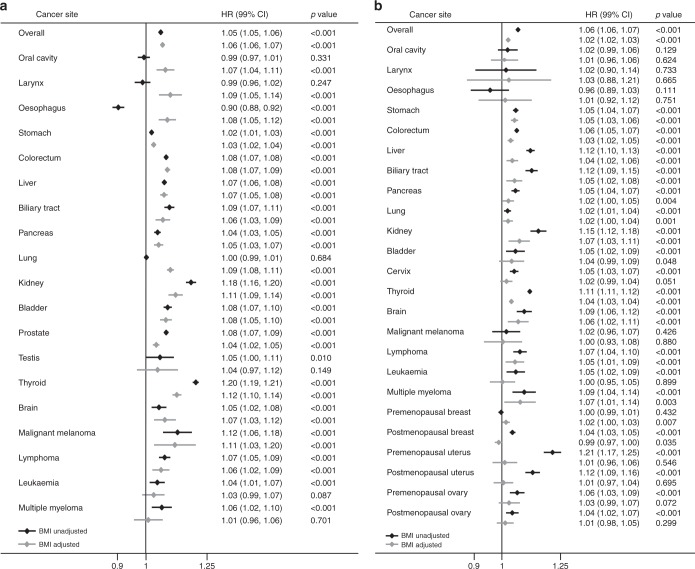
Forest plot of the HR for each cancer across WC quintiles before and after BMI adjustment, from models (adjusted for age, smoking status (3 categories), alcohol drinking (3 categories), physical activity (2 categories), co-morbidities (e.g., hypertension, type 2 diabetes, and hyperlipidemia; all are 2 categories, respectively), and socioeconomic status (2 categories)) fitted as a linear effect. **a** Male. **b** Female. *HR* hazard ratio, *WC* waist circumference, *BMI* body mass index

Table 2 shows the HRs for developing cancers compared with individuals with the lowest WC (quintile 1), after controlling for confounding factors including the BMI. Figure 1 also depicts the forest plot for each cancer across WC quintiles before and after BMI adjustment. The data confirmed that in the Korean population, central obesity strongly increased the risk of cancer in the stomach, colorectum, hepatobiliary system, kidney, thyroid, brain, and lymphoma in a dose-dependent manner (*P*_trend_ < 0.001 for both sexes). Gender specific differences in significance were notable in some cancers, with more robust associations in males for cancers of the head and neck, oesophagus, pancreas, lung, bladder and skin, and more robust correlations in females for multiple myeloma. There were also significant dose-dependent relationships between WC and cancers of the prostate (*P*_trend_ < 0.001) and breast (premenopausal, *P*_trend_ = 0.007; postmenopausal, inversely *P*_trend_ = 0.035), but associations with other genital tract cancers were not reach the significant. The BMI did not affect or attenuated the WC-cancer association for most cancers. However, for cancers of the oral cavity, larynx, oesophagus, and lung in male (all *P*_trend_ < 0.001) and premenopausal breast (*P*_trend_ = 0.007), the BMI adjustment reversed the association toward being positive.

**Table 2:** Multivariate hazard ratio and 99% confidence interval for developing cancers by waist circumference quintile

Cancer site	Male						Female					
	Q1	Q2	Q3	Q4	Q5	*P*_trend_	Q1	Q2	Q3	Q4	Q5	*P*_trend_
Overall												
No. cancer	52,220	72,638	76,014	94,007	90,321		49,394	69,656	67,691	93,804	104,126	
HR^a^ (99% CI)	1	1.09 (1.07–1.11)	1.14 (1.12–1.15)	1.19 (1.17–1.20)	1.26 (1.24–1.28)	<0.001	1	1.20 (1.18–1.22)	1.27 (1.25–1.29)	1.30 (1.28–1.32)	1.35 (1.33–1.37)	<0.001
HR^b^ (99% CI)	1	1.10 (1.08–1.12)	1.15 (1.14–1.17)	1.21 (1.19–1.23)	1.30 (1.27–1.33)	<0.001	1	1.15 (1.13–1.17)	1.19 (1.17–1.21)	1.17 (1.15–1.19)	1.14 (1.12–1.17)	<0.001
Oral cavity												
No. cancer	1245	1619	1560	1829	1656		371	533	525	784	859	
HR^a^ (99% CI)	1	1.02 (0.92–1.12)	0.98 (0.89–1.09)	0.98 (0.89–1.08)	0.98 (0.89–1.08)	0.331	1	1.11 (0.93–1.33)	1.12 (0.94–1.34)	1.16 (0.98–1.38)	1.12 (0.94–1.34)	0.129
HR^b^ (99% CI)	1	1.13 (1.02–1.25)	1.16 (1.04–1.30)	1.23 (1.10–1.39)	1.38 (1.20–1.59)	<0.001	1	1.10 (0.92–1.31)	1.09 (0.90–1.32)	1.12 (0.92–1.36)	1.06 (0.84–1.33)	0.624
Larynx												
No. cancer	759	913	874	1094	970		31	49	67	64	105	
HR^a^ (99% CI)	1	0.98 (0.86–1.11)	0.93 (0.82–1.06)	0.98 (0.86–1.11)	0.93 (0.82–1.06)	0.247	1	1.12 (0.62–2.04)	1.46 (0.83–2.59)	0.91 (0.51–1.64)	1.24 (0.71–2.17)	0.733
HR^b^ (99% CI)	1	1.11 (0.97–1.27)	1.15 (1.00–1.33)	1.31 (1.12–1.53)	1.44 (1.20–1.73)	<0.001	1	1.15 (0.63–2.11)	1.52 (0.83–2.78)	0.96 (0.50–1.85)	1.35 (0.65–2.79)	0.665
Oesophagus												
No. cancer	1522	1773	1484	1713	1475		92	132	128	190	237	
HR^a^ (99% CI)	1	0.93 (0.85–1.02)	0.77 (0.70–0.85)	0.74 (0.67–0.81)	0.68 (0.61–0.75)	<0.001	1	0.97 (0.68–1.38)	0.87 (0.61–1.24)	0.83 (0.59–1.16)	0.85 (0.61–1.19)	0.111
HR^b^ (99% CI)	1	1.17 (1.07–1.29)	1.12 (1.01–1.25)	1.25 (1.11–1.40)	1.47 (1.28–1.69)	<0.001	1	1.04 (0.73–1.49)	0.98 (0.67–1.43)	0.98 (0.66–1.43)	1.10 (0.71–1.72)	0.751
Stomach												
No. cancer	13,544	17,782	18,213	21,898	20,122		4069	6402	6685	10,584	13,294	
HR^a^ (99% CI)	1	1.04 (1.01–1.07)	1.06 (1.03–1.09)	1.07 (1.04–1.10)	1.09 (1.05–1.12)	<0.001	1	1.13 (1.07–1.19)	1.14 (1.08–1.20)	1.20 (1.14–1.26)	1.28 (1.22–1.34)	<0.001
HR^b^ (99% CI)	1	1.05 (1.02–1.08)	1.07 (1.04–1.11)	1.10 (1.06–1.14)	1.12 (1.08–1.17)	<0.001	1	1.13 (1.07–1.19)	1.13 (1.07–1.20)	1.19 (1.12–1.26)	1.26 (1.18–1.34)	<0.001
Colorectum												
No. cancer	10,803	15,809	17,189	21,888	21,324		6283	9785	10,577	15,846	19,893	
HR^a^ (99% CI)	1	1.13 (1.10–1.17)	1.21 (1.18–1.25)	1.29 (1.25–1.33)	1.37 (1.33–1.41)	<0.001	1	1.15 (1.10–1.19)	1.22 (1.17–1.27)	1.23 (1.18–1.28)	1.32 (1.27–1.37)	<0.001
HR^b^ (99% CI)	1	1.14 (1.10–1.18)	1.22 (1.18–1.27)	1.31 (1.26–1.36)	1.40 (1.34–1.50)	<0.001	1	1.12 (1.07–1.17)	1.17 (1.12–1.22)	1.15 (1.10–1.21)	1.19 (1.13–1.26)	<0.001
Liver^c^												
No. cancer	5857	8458	8659	10,524	10,939		1410	2378	2731	4484	6233	
HR^a^ (99% CI)	1	1.14 (1.10–1.20)	1.18 (1.13–1.23)	1.21 (1.16–1.26)	1.37 (1.31–1.43)	<0.001	1	1.20 (1.10–1.31)	1.31 (1.21–1.43)	1.41 (1.30–1.53)	1.61 (1.49–1.75)	<0.001
HR^b^ (99% CI)	1	1.15 (1.10–1.20)	1.18 (1.12–1.24)	1.21 (1.15–1.28)	1.38 (1.30–1.47)	<0.001	1	1.11 (1.02–1.22)	1.16 (1.06–1.27)	1.18 (1.08–1.29)	1.22 (1.10–1.35)	<0.001
Biliary tract^d^												
No. cancer	1354	1945	2218	2870	2768		584	1085	1392	2489	3476	
HR^a^ (99% CI)	1	1.15 (1.05–1.26)	1.29 (1.18–1.41)	1.38 (1.27–1.51)	1.43 (1.31–1.56)	<0.001	1	1.20 (1.05–1.37)	1.37 (1.21–1.56)	1.51 (1.33–1.70)	1.65 (1.46–1.86)	<0.001
HR^b^ (99% CI)	1	1.12 (1.02–1.23)	1.22 (1.11–1.35)	1.29 (1.16–1.42)	1.28 (1.14–1.45)	<0.001	1	1.12 (0.98–1.28)	1.22 (1.07–1.39)	1.27 (1.11–1.45)	1.25 (1.08–1.45)	<0.001
Pancreas												
No. cancer	3712	5150	5573	6820	6381		1649	2759	2958	4798	6127	
HR^a^ (99% CI)	1	1.08 (1.02–1.14)	1.15 (1.09–1.22)	1.18 (1.12–1.24)	1.19 (1.13–1.26)	<0.001	1	1.17 (1.08–1.27)	1.19 (1.10–1.29)	1.25 (1.16–1.35)	1.30 (1.20–1.40)	<0.001
HR^b^ (99% CI)	1	1.08 (1.02–1.15)	1.16 (1.09–1.23)	1.19 (1.11–1.27)	1.21 (1.12–1.31)	<0.001	1	1.14 (1.05–1.24)	1.13 (1.04–1.23)	1.16 (1.06–1.26)	1.16 (1.05–1.27)	0.004
Lung												
No. cancer	8160	9501	9453	11,471	10,413		2173	3622	3894	6129	7317	
HR^a^ (99% CI)	1	0.98 (0.94–1.01)	0.98 (0.94–1.02)	1.00 (0.96–1.04)	0.99 (0.95–1.03)	0.684	1	1.15 (1.07–1.23)	1.15 (1.08–1.24)	1.17 (1.09–1.25)	1.15 (1.08–1.23)	<0.001
HR^b^ (99% CI)	1	1.09 (1.05–1.13)	1.17 (1.12–1.22)	1.29 (1.23–1.35)	1.44 (1.36–1.52)	<0.001	1	1.15 (1.07–1.23)	1.16 (1.07–1.24)	1.17 (1.09–1.26)	1.16 (1.06–1.26)	0.001
Kidney												
No. cancer	1102	1990	2344	3110	3450		417	698	889	1339	1865	
HR^a^ (99% CI)	1	1.36 (1.23–1.50)	1.58 (1.44–1.73)	1.75 (1.60–1.92)	2.10 (1.92–2.30)	<0.001	1	1.28 (1.09–1.51)	1.63 (1.39–1.91)	1.65 (1.42–1.91)	1.91 (1.64–2.22)	<0.001
HR^b^ (99% CI)	1	1.27 (1.15–1.40)	1.41 (1.28–1.57)	1.50 (1.35–1.67)	1.66 (1.47–1.88)	<0.001	1	1.19 (1.01–1.40)	1.42 (1.21–1.68)	1.36 (1.15–1.60)	1.40 (1.16–1.69)	<0.001
Bladder												
No. cancer	2135	3017	3159	4119	4208		259	489	572	939	1354	
HR^a^ (99% CI)	1	1.14 (1.06–1.23)	1.18 (1.10–1.27)	1.28 (1.20–1.38)	1.41 (1.31–1.51)	<0.001	1	1.20 (0.99–1.47)	1.22 (1.01–1.49)	1.21 (1.01–1.46)	1.33 (1.11–1.60)	<0.001
HR^b^ (99% CI)	1	1.14 (1.05–1.23)	1.17 (1.08–1.27)	1.26 (1.16–1.37)	1.38 (1.25–1.52)	<0.001	1	1.19 (0.97–1.45)	1.20 (0.98–1.47)	1.17 (0.96–1.43)	1.27 (1.01–1.59)	0.048
Thyroid												
No. cancer	3499	6097	6525	8096	7967		19,022	25,509	23,095	29,019	27,486	
HR^a^ (99% CI)	1	1.45 (1.37–1.53)	1.71 (1.62–1.80)	1.94 (1.84–2.05)	2.27 (2.15–2.39)	<0.001	1	1.38 (1.35–1.41)	1.57 (1.53–1.61)	1.64 (1.59–1.68)	1.62 (1.58–1.67)	<0.001
HR^b^ (99% CI)	1	1.33 (1.25–1.40)	1.49 (1.40–1.58)	1.60 (1.50–1.70)	1.68 (1.56–1.81)	<0.001	1	1.27 (1.23–1.30)	1.36 (1.32–1.39)	1.33 (1.29–1.37)	1.16 (1.12–1.21)	<0.001
Brain												
No. cancer	783	1014	996	1215	1173		600	855	930	1345	1706	
HR^a^ (99% CI)	1	1.03 (0.91–1.16)	1.04 (0.92–1.18)	1.10 (0.98–1.24)	1.22 (1.07–1.37)	<0.001	1	1.13 (0.98–1.29)	1.27 (1.11–1.46)	1.29 (1.13–1.47)	1.45 (1.27–1.66)	<0.001
HR^b^ (99% CI)	1	1.06 (0.93–1.20)	1.09 (0.95–1.25)	1.18 (1.02–1.36)	1.34 (1.12–1.60)	<0.001	1	1.10 (0.96–1.27)	1.22 (1.05–1.14)	1.21 (1.04–1.41)	1.32 (1.11–1.58)	<0.001
Malignant melanoma												
No. cancer	153	230	311	356	353		181	207	253	410	509	
HR^a^ (99% CI)	1	1.16 (0.89–1.52)	1.55 (1.20–2.01)	1.50 (1.17–1.93)	1.64 (1.27–2.12)	<0.001	1	0.79 (0.60–1.02)	0.89 (0.69–1.16)	0.93 (0.73–1.18)	0.95 (0.75–1.07)	0.426
HR^b^ (99% CI)	1	1.15 (0.87–1.51)	1.52 (1.15–2.01)	1.45 (1.01–1.95)	1.56 (1.10–2.21)	<0.001	1	0.78 (0.59–1.01)	0.87 (0.67–1.15)	0.90 (0.69–1.18)	0.90 (0.66–1.23)	0.880
Lymphoma												
No. cancer	1213	1648	1716	2147	1995		690	1178	1134	1686	1918	
HR^a^ (99% CI)	1	1.07 (0.97–1.18)	1.13 (1.03–1.25)	1.23 (1.12–1.35)	1.31 (1.19–1.44)	<0.001	1	1.34 (1.19–1.52)	1.35 (1.19–1.53)	1.42 (1.25–1.60)	1.46 (1.29–1.66)	<0.001
HR^b^ (99% CI)	1	1.05 (0.95–1.16)	1.10 (0.99–1.23)	1.18 (1.05–1.32)	1.23 (1.07–1.41)	<0.001	1	1.31 (1.16–1.49)	1.30 (1.13–1.48)	1.34 (1.17–1.54)	1.35 (1.15–1.58)	<0.001
Leukaemia												
No. cancer	755	1001	996	1203	1085		434	620	615	855	1012	
HR^a^ (99% CI)	1	1.05 (0.92–1.18)	1.07 (0.95–1.22)	1.13 (1.00–1.28)	1.18 (1.04–1.34)	<0.001	1	1.17 (0.99–1.38)	1.23 (1.04–1.45)	1.22 (1.04–1.44)	1.31 (1.11–1.54)	<0.001
HR^b^ (99% CI)	1	1.03 (0.90–1.17)	1.04 (0.91–1.20)	1.09 (0.94–1.26)	1.11 (0.93–1.33)	0.087	1	1.11 (0.94–1.31)	1.12 (0.94–1.34)	1.07 (0.89–1.29)	1.06 (0.85–1.31)	0.899
Multiple myeloma												
No. cancer	405	517	576	707	679		176	304	403	653	796	
HR^a^ (99% CI)	1	1.01 (0.85–1.20)	1.12 (0.95–1.33)	1.17 (0.99–1.37)	1.24 (1.05–1.47)	<0.001	1	1.17 (0.92–1.49)	1.43 (1.13–1.82)	1.48 (1.19–1.86)	1.48 (1.18–1.86)	<0.001
HR^b^ (99% CI)	1	0.95 (0.80–1.14)	1.01 (0.84–1.22)	1.01 (0.83–1.23)	1.00 (0.79–1.26)	0.701	1	1.14 (0.89–1.47)	1.38 (1.08–1.77)	1.40 (1.10–1.80)	1.36 (1.03–1.80)	0.003
Prostate												
No. cancer	6531	9295	10,375	13,359	12,932							
HR^a^ (99% CI)	1	1.15 (1.10–1.20)	1.25 (1.20–1.30)	1.33 (1.27–1.38)	1.37 (1.32–1.43)	<0.001	—					
HR^b^ (99% CI)	1	1.09 (1.05–1.14)	1.16 (1.10–1.21)	1.19 (1.14–1.25)	1.17 (1.11–1.23)	<0.001	—					
Testis												
No. cancer	227	293	298	346	341							
HR^a^ (99% CI)	1	1.01 (0.80–1.27)	1.07 (0.85–1.34)	1.08 (0.87–1.36)	1.23 (0.98–1.55)	0.010	—					
HR^b^ (99% CI)	1	1.00 (0.79–1.27)	1.05 (0.82–1.36)	1.06 (0.81–1.40)	1.20 (0.86–1.67)	0.149	—					
Breast-premenopausal												
No. cancer							8897	9958	7083	6775	4479	
HR^a^ (99% CI)	—						1	1.03 (0.99–1.08)	1.03 (0.99–1.08)	1.02 (0.98–1.07)	0.98 (0.93–1.03)	0.432
HR^b^ (99% CI)	—						1	1.05 (1.01–1.09)	1.06 (1.02–1.11)	1.07 (1.02–1.13)	1.05 (0.98–1.13)	0.007
Breast-postmenopausal												
No. cancer							2942	5674	6778	10,365	11,884	
HR^a^ (99% CI)	—						1	1.00 (0.94–1.06)	1.04 (0.99–1.11)	1.08 (1.02–1.14)	1.15 (1.09–1.22)	<0.001
HR^b^ (99% CI)	—						1	0.95 (0.89–1.01)	0.96 (0.90–1.02)	0.95 (0.89–1.00)	0.93 (0.87–0.99)	0.035
Uterus-premenopausal												
No. cancer							692	795	580	684	824	
HR^a^ (99% CI)	—						1	1.11 (0.97–1.27)	1.15 (0.99–1.33)	1.39 (1.21–1.61)	2.31 (2.01–2.66)	<0.001
HR^b^ (99% CI)	—						1	0.95 (0.83–1.09)	0.87 (0.74–1.02)	0.93 (0.78–1.09)	1.13 (0.92–1.39)	0.546
Uterus-postmenopausal												
No. cancer							408	834	1024	1612	2273	
HR^a^ (99% CI)	—						1	1.05 (0.90–1.23)	1.13 (0.97–1.32)	1.19 (1.03–1.38)	1.55 (1.34–1.78)	<0.001
HR^b^ (99% CI)	—						1	0.96 (0.82–1.12)	0.95 (0.82–1.12)	0.93 (0.79–1.08)	1.02 (0.86–1.21)	0.695
Ovary-premenopausal												
No. cancer							1269	1261	886	882	799	
HR^a^ (99% CI)	—						1	0.99 (0.89–1.10)	1.00 (0.90–1.13)	1.05 (0.93–1.18)	1.35 (1.19–1.52)	<0.001
HR^b^ (99% CI)	—						1	0.97 (0.87–1.08)	0.97 (0.85–1.10)	0.99 (0.86–1.14)	1.23 (1.03–1.47)	0.072
Ovary-postmenopausal												
No. cancer							597	1102	1447	2164	2748	
HR^a^ (99% CI)	—						1	0.95 (0.83–1.08)	1.06 (0.93–1.20)	1.02 (0.90–1.15)	1.14 (1.01–1.28)	<0.001
HR^b^ (99% CI)	—						1	0.93 (0.81–1.06)	1.02 (0.89–1.16)	0.96 (0.84–1.09)	1.03 (0.89–1.20)	0.299
Cervix												
No. cancer							2459	2986	2693	3587	4117	
HR^a^ (99% CI)	—						1	1.08 (1.01–1.16)	1.11 (1.03–1.19)	1.13 (1.05–1.21)	1.24 (1.15–1.33)	<0.001
HR^b^ (99% CI)	—						1	1.05 (0.98–1.13)	1.05 (0.97–1.14)	1.05 (0.96–1.14)	1.10 (1.00–1.22)	0.051

*HR* hazard ratio, *CI* confidence interval.

^a^Adjusted for age, smoking status (3 categories), alcohol drinking (3 categories), physical activity (2 categories), co-morbidities (e.g., hypertension, type 2 diabetes, and hyperlipidemia; all are 2 categories, respectively), and socioeconomic status (2 categories).

^b^Adjusted for body mass index (continuous) additionally.

^c^Includes intrahepatic biliary tract cancer.

^d^Includes gallbladder cancer and extrahepatic biliary tract cancer

Selected sensitivity analyses are shown in the supplementary tables. Because smoking has an inverse relationship with obesity and is a well-known risk factor for many cancers, we analysed data for those who had never smoked. The overall trend and its significance involving the WC-cancer association remained in smoking-related cancer, while the significance diminished in some cancers (Table S1). To examine the effect of preclinical cancers that may cause weight loss and thus bias the association between obesity and cancer, we repeated the analyses after excluding cancer occurring in patients within the first 2 years of follow up, but found no significant difference (Table S2).

## Discussion

Continuous updates of the scientific literature have supported the association between high WC and the risk of cancer. In a single dataset, we determined the associations between WC and the incidence of cancer, when considering potential confounders including the BMI. To the best of our knowledge, this is the largest study to estimate the effects of WC-cancer associations across a range of sites. In addition, studies on this issue have been conducted primarily in Western populations. Asians, including Koreans, tend to have relatively small body frames and condensed body fat. Compared with Caucasians, they have a higher body fat percentage for a given BMI^29^. Moreover, they have higher amounts of abdominal adipose tissue^31,32^ and lower muscle mass for a given BMI, leading to a greater tendency toward central obesity and to more susceptible to insulin resistance^33,34^. Thus, the effects of abdominal obesity on the development of cancer in Asians might differ from those in Caucasians.

Several mechanisms have been proposed to explain links between adiposity and increased cancer risk involving sex hormone metabolism, insulin and IFG signalling, and adipokine pathophysiology^35^. However, excess body fat is a heterogeneous condition in which individuals with similar BMIs may have distinct cancer risks. Our results provide a potential explanation for the risk differential that persists after accounting for BMI. Few studies have conducted further adjustments between the central adiposity index and BMI to clarify their independent roles in the risk of cancers.

It is currently recognised that a proportion of obese individuals might not be at an increased risk for metabolic complications of obesity. Recent studies have suggested a correlation between metabolic health and cancer outcomes^36,37^. Metabolically active visceral fat releases substantial amounts of growth factors, inflammatory markers, free fatty acids, and locally produced oestrogen and adipokines, which might contribute to the development of cancer^38^. However, WC not only distinguishes lean from adipose tissue, but reflects adipose tissues in both subcutaneous and visceral areas, which obscure any separate roles of compartments in determining the cancer risk. The most comprehensive quantitative modalities will come from large-scale imaging (i.e., abdominal fat computed tomography and dual-energy X-ray absorptiometry) projects, which should result in the identification of dominant mechanistic pathways.

We identified several cancers related to central obesity that were independent of general adiposity. Overall, this finding was consistent with those from recent studies of cancers of the gastrointestinal tract^10,39,40^, lung^14^, hepato-pancreato-biliary system^13,41–43^, head and neck^17^, and kidney^40^. Cancers of the thyroid^16^, prostate^20,44^, bladder^45^, and skin^46^, which had conflicting or non-significant results, also had significantly positive dose-dependent relationships with increasing WC. Non-significant results in prior studies might be simply due to relatively small sample sizes, as their associations were similar to ours, and the associations reached significance before adjusting for the BMI. However, for prostate cancer, we were unable to explain the disagreement with previous results, because the association between body adiposity and incident prostate cancer depends mainly on the stage or subtype of prostate cancer^20^. Prostate cancer has a large clinical heterogeneity, which ranges from microscopic, well-differentiated indolent tumours to aggressive and lethal diseases. However, in the present study, we could not obtain this detailed clinical information.

We did find a new association between WC and lymphoma/brain tumours, which has not been previously reported because the BMI was not adjusted in previous studies^47,48^. In contrast, we could not find an association for leukaemia that has previously been reported to be associated with central adiposity^49^. For multiple myeloma, while a recent Mendelian randomisation study reported a non-significant (*p* = 0.06) reverse association of the BMI-adjusted WC^50^, we found a positive association in female (*P*_trend_ = 0.003). Given the largely unknown etiology and the heterogeneous entity of these malignancies, further studies are warranted to clarify their associations with central adiposity.

We found differential associations of WC by sex with the risks of some cancers, but this might have been largely because of the differences in sample size (i.e., cancers of larynx, oesophagus, and bladder). Besides the sample size gap, a male-specific association with malignant melanoma was also found. Malignant melanoma is the most aggressive form of skin cancer, which is considered the fastest growing cancer^51^. Overall, a link between malignant melanoma and general adiposity remains unclear^52^, although a recent meta-analysis reported a male-specific effect^53^. To the best our knowledge, only a single study investigated the impact of central obesity when considering the BMI, with no convincing associations reported^46^. A possible explanation is that increased body surface in males may simply denote a larger surface at risk for sunlight exposure, thus providing an increased association with the incidences of melanoma^53^. However, our sensitivity analyses showed that the positive association between WC and melanoma in males was largely affected by the smoking status, and not by the BMI. Smoking may be at least a strong modifier in the WC-melanoma associations, even though it is not a risk factor for melanoma^54^. There has also been growing evidence that smoking is associated with abdominal fat accumulation^55,56^.

In the present study, positive associations were mostly attenuated after BMI adjustment; however, notable reversions toward positive associations were found in cancers of the oral cavity, larynx, oesophagus, and lung. While the mean BMI values are generally lower in smokers than in non-smokers^57^, cigarette smoking has been positively associated with central adiposity^55,58^. Our additional analyses in non-smokers revealed an unaltered direction or significance of association. This means that metabolic derangements, represented by central adiposity, are still responsible for increased risk in such cancers, even when excluding the effect of smoking. In this regard, obtaining a WC measurement together with BMI may provide essential information that might not be feasible when assessing each parameter separately.

In a similar manner, it was noteworthy that among oestrogen-driven cancers, BMI-adjusted WC predicted the risk of premenopausal breast cancer. Before menopause, plasma levels of oestrogen were not directly related to general adiposity, and obese premenopausal females had lower estradiol levels because they are more likely to have anovulatory cycles^59^. Thus, the positive association with WC in this study cannot be explained by the sex hormone hypothesis, which was supported by the finding that central obesity was associated with an increased risk of oestrogen receptor-negative breast cancer in premenopausal women^60^. Central obesity is a well-known indicator of hyperinsulinemia and higher levels of IGF-1 that are related to premenopausal breast cancer risk^61^. Overall, our results indicated that metabolic conditions may be more important than hormonal mechanisms in premenopausal breast cancer.

Occult cancers are an important type of cancer to be considered. To eliminate bias, we performed sensitivity analyses excluding an incident cancer within the initial 2 years of follow up; however, we could not find any significant differences. We assumed that this was because many of the occult cancer patients were diagnosed and excluded through their health status examination near the baseline. In addition, the general perception is that obesity does not initiate cancer, but rather promotes cancer in clinical presentations over several years. The precise time lag between development and duration of obesity and the occurrence of cancer is still not well established.

There are some limitations in this study. Our study was confined to individuals who took a health examination, so those without WC data were excluded, introducing the possibility of selection bias. Based on the 2009 Korea National Health and Nutrition Examination Survey data^62^, a prevalence of obesity/smoker was 35.8%/47% for male and 26%/7.1% for female, respectively: these are comparable to our results (37.3%/44.9% for male and 26.6%/4.1% for female). Moreover, our main conclusions are unlikely to have been seriously affected, because we investigated the WC-cancer relation itself. A second limitation was the potential for residual confounding that we could not consider. Our outcome data were insufficiently detailed to explore potential differences between cancer subtypes, in particular for oesophageal cancer^6^, lung cancer^63^, breast cancer^64^, prostate cancer^20^, lymphoma^65^, and colon cancer^66^. We also had no detailed information on critical risk factors for some cancers, such as female reproductive factors for oestrogen-dependent tumours, viral hepatitis information for liver cancer, ultraviolet exposures for melanoma, and amounts or forms of tobacco consumption for lung cancer. Mendelian randomisation may offer a solution to the problem of residual confounding, under certain conditions^67^. A final limitation was the assumption of an unchangeable obesity index. We assessed only baseline measures, so these single measurements may not have reflected changes that occurred during the follow up. Previous studies^68,69^ also emphasised the importance of time duration with a high BMI and its association with cancer development, in a similar manner to other diseases^70^. Longer follow up with sequential measurements is needed to more fully support a causal association.

Despite these limitations, our study has methodological strengths. As a longitudinal, nationwide, population-based cohort study, the size of the dataset was close to the entire population of adult Koreans. Compulsory health examinations also allowed us to use direct anthropometric measurements, which are preferred over self-reported data^71^. To date, no other study has used direct measurements of WC in such a large sample size for a wide range of cancer sites. Our results provide evidence on the independent role of central obesity as a predictor for cancer incidence, even in a relatively lean Asian population. However, this result is only indirect evidence that the interventional effect of WC reduction will decrease the risk of cancer. Heterogeneity in the effects of BMI also suggests that there are different mechanisms or combinations of mechanisms associated with different sites. Integration of experimental research on underlying mechanisms linking central obesity to the identified cancers in our study is necessary to confirm our conclusions.

## Electronic supplementary material

Supplemental tables 1-2

## Availability of data and material

The data were provided by National Health Insurance Sharing Service. These are third party data. We did not have any special access privileges that others would not have. Anyone can submit a research proposal online (https://nhiss.nhis.or.kr/bd/ab/bdaba021eng.do). If the proposal is accepted by an evaluation committee of NHIC, researcher would access the de-identified NHIC dataset after paying some fee. Permission for the use of health check-up data was granted by the NHIC (NHIS-2018-1-174).
