# Supplementary material for: Waist circumference and risk of 23 site-specific cancers: a population-based cohort study of Korean adults
Source: Br J Cancer. 2018 Oct 17;119(8):1018–27. doi: 10.1038/s41416-018-0214-7 (PMC6203821; doi:10.1038/s41416-018-0214-7)
Supplement: Supplementary file 1 — Supplemental tables 1-2 [file 41416_2018_214_MOESM1_ESM.docx]

**Table S1.** Association between waist circumference and cancer in the entire study population and individuals who never smoked

|  |  | Male |  |  |  |  |  | Female |  |  |  |  |  |
| --- | --- | --- | --- | --- | --- | --- | --- | --- | --- | --- | --- | --- | --- |
| Cancer site | | Q1 | Q2 | Q3 | Q4 | Q5 | *P*_trend_ | Q1 | Q2 | Q3 | Q4 | Q5 | *P*_trend_ |
| *Smoking-related cancers* | |  |  |  |  |  |  |  |  |  |  |  |  |
| No. cancer in oral cavity | | 298/1,245 | 423/1,619 | 413/1,560 | 536/1,829 | 509/1,656 |  | 338/371 | 489/533 | 498/525 | 735/784 | 808/859 |  |
|  | Entire | 1 | 1.13 (1.02–1.25) | 1.16 (1.04–1.30) | 1.23 (1.10–1.39) | 1.38 (1.20–1.59) | <0.001 | 1 | 1.10 (0.92–1.31) | 1.09 (0.90–1.32) | 1.12 (0.92–1.36) | 1.06 (0.84–1.33) | 0.624 |
|  | Never smoked | 1 | 1.18 (0.96–1.44) | 1.18 (0.95–1.47) | 1.35 (1.08–1.69) | 1.51 (1.15–1.97) | <0.001 | 1 | 1.09 (0.90–1.31) | 1.11 (0.91–1.36) | 1.12 (0.92–1.38) | 1.07 (0.84–1.36) | 0.489 |
| No. cancer in larynx | | 126/759 | 160/913 | 175/874 | 243/1,094 | 236/970 |  | 21/31 | 37/49 | 58/67 | 55/64 | 89/105 |  |
|  | Entire | 1 | 1.11 (0.97–1.27) | 1.15 (1.00–1.33) | 1.31 (1.12–1.53) | 1.44 (1.20–1.73) | <0.001 | 1 | 1.15 (0.63–2.11) | 1.52 (0.83–2.78) | 0.96 (0.50–1.85) | 1.35 (0.65–2.79) | 0.665 |
|  | Never smoked | 1 | 1.17 (0.85–1.61) | 1.36 (0.98–1.90) | 1.70 (1.21–2.40) | 2.05 (1.37–3.06) | <0.001 | 1 | 1.21 (0.59–2.48) | 1.79 (0.89–3.61) | 1.10 (0.52–2.34) | 1.46 (0.63–3.36) | 0.574 |
| No. cancer in esophagus | | 313/1,522 | 391/1,773 | 383/1,484 | 467/1,713 | 463/1,475 |  | 72/92 | 101/132 | 116/128 | 163/190 | 220/237 |  |
|  | Entire | 1 | 1.17 (1.07–1.29) | 1.12 (1.01–1.25) | 1.25 (1.11–1.40) | 1.47 (1.28–1.69) | <0.001 | 1 | 1.04 (0.73–1.49) | 0.98 (0.67–1.43) | 0.98 (0.66–1.43) | 1.10 (0.71–1.72) | 0.751 |
|  | Never smoked | 1 | 1.16 (0.95–1.42) | 1.21 (0.97–1.51) | 1.33 (1.06–1.68) | 1.65 (1.26–2.16) | <0.001 | 1 | 0.96 (0.64–1.43) | 1.02 (0.68–1.55) | 0.94 (0.61–1.43) | 1.11 (0.68–1.79) | 0.629 |
| No. cancer in stomach | | 3,751/13,544 | 5,047/17,782 | 5,448/18,213 | 6,588/21,898 | 6,485/20,122 |  | 3,770/4,069 | 6,009/6,402 | 6,307/6,685 | 10,073/10,584 | 12,650/13,294 |  |
|  | Entire | 1 | 1.05 (1.02–1.08) | 1.07 (1.04–1.11) | 1.10 (1.06–1.14) | 1.12 (1.08–1.17) | <0.001 | 1 | 1.13 (1.07–1.19) | 1.13 (1.07–1.20) | 1.19 (1.12–1.26) | 1.26 (1.18–1.34) | <0.001 |
|  | Never smoked | 1 | 1.04 (0.98–1.10) | 1.08 (1.02–1.15) | 1.08 (1.01–1.15) | 1.12 (1.04–1.21) | <0.001 | 1 | 1.12 (1.06–1.19) | 1.13 (1.07–1.20) | 1.19 (1.13–1.26) | 1.27 (1.19–1.35) | <0.001 |
| No. cancer in colorectum | | 3,135/10,803 | 4,704/15,809 | 5,429/17,189 | 7,100/21,888 | 7,095/21,324 |  | 5,820/6,283 | 9,201/9,785 | 10,047/10,577 | 15,120/15,846 | 18,899/19,893 |  |
|  | Entire | 1 | 1.14 (1.10–1.18) | 1.22 (1.18–1.27) | 1.31 (1.26–1.36) | 1.40 (1.34–1.50) | <0.001 | 1 | 1.12 (1.07–1.17) | 1.17 (1.12–1.22) | 1.15 (1.10–1.21) | 1.19 (1.13–1.26) | <0.001 |
|  | Never smoked | 1 | 1.14 (1.08–1.22) | 1.27 (1.20–1.36) | 1.37 (1.28–1.46) | 1.44 (1.34–1.56) | <0.001 | 1 | 1.12 (1.07–1.17) | 1.17 (1.12–1.23) | 1.16 (1.10–1.21) | 1.20 (1.13–1.26) | <0.001 |
| No. cancer in liver | | 1,687/5,857 | 2,495/8,458 | 2,650/8,659 | 3,317/10,524 | 3,646/10,939 |  | 1,284/1,410 | 2,206/2,378 | 2,558/2,731 | 4,249/4,484 | 5,907/6,233 |  |
|  | Entire | 1 | 1.15 (1.10–1.20) | 1.18 (1.12–1.24) | 1.21 (1.15–1.28) | 1.38 (1.30–1.47) | <0.001 | 1 | 1.11 (1.02–1.22) | 1.16 (1.06–1.27) | 1.18 (1.08–1.29) | 1.22 (1.10–1.35) | <0.001 |
|  | Never smoked | 1 | 1.15 (1.06–1.25) | 1.20 (1.10–1.31) | 1.25 (1.14–1.38) | 1.47 (1.32–1.63) | <0.001 | 1 | 1.11 (1.02–1.22) | 1.16 (1.06–1.28) | 1.19 (1.08–1.31) | 1.23 (1.11–1.36) | <0.001 |
| No. cancer in pancreas | | 1,061/3,712 | 1,469/5,150 | 1,674/5,573 | 2,115/6,820 | 1,983/6,381 |  | 1,534/1,649 | 2,595/2,759 | 2,803/2,958 | 4,559/4,798 | 5,808/6,127 |  |
|  | Entire | 1 | 1.08 (1.02–1.15) | 1.16 (1.09–1.23) | 1.19 (1.11–1.27) | 1.21 (1.12–1.31) | <0.001 | 1 | 1.14 (1.05–1.24) | 1.13 (1.04–1.23) | 1.16 (1.06–1.26) | 1.16 (1.05–1.27) | 0.004 |
|  | Never smoked | 1 | 1.06 (0.95–1.18) | 1.17 (1.04–1.30) | 1.21 (1.08–1.36) | 1.19 (1.04–1.37) | <0.001 | 1 | 1.14 (1.04–1.24) | 1.13 (1.03–1.23) | 1.16 (1.06–1.26) | 1.16 (1.05–1.28) | 0.004 |
| No. cancer in lung | | 1,703/8,160 | 2,166/9,501 | 2,194/9,453 | 2,746/11,471 | 2,573/10,413 |  | 1,957/2,173 | 3,361/3,622 | 3,620/3,894 | 5,705/6,129 | 6,784/7,317 |  |
|  | Entire | 1 | 1.09 (1.05–1.13) | 1.17 (1.12–1.22) | 1.29 (1.23–1.35) | 1.44 (1.36–1.52) | <0.001 | 1 | 1.15 (1.07–1.23) | 1.16 (1.07–1.24) | 1.17 (1.09–1.26) | 1.16 (1.06–1.26) | 0.001 |
|  | Never smoked | 1 | 1.13 (1.04–1.23) | 1.20 (1.09–1.31) | 1.32 (1.20–1.46) | 1.49 (1.32–1.67) | <0.001 | 1 | 1.16 (1.08–1.25) | 1.16 (1.08–1.26) | 1.18 (1.09–1.27) | 1.16 (1.06–1.26) | 0.004 |
| No. cancer in kidney | | 361/1,102 | 582/1,990 | 768/2,344 | 981/3,110 | 1,082/3,450 |  | 389/417 | 670/698 | 853/889 | 1,273/1,339 | 1,777/1,865 |  |
|  | Entire | 1 | 1.27 (1.15–1.40) | 1.41 (1.28–1.57) | 1.50 (1.35–1.67) | 1.66 (1.47–1.88) | <0.001 | 1 | 1.19 (1.01–1.40) | 1.42 (1.21–1.68) | 1.36 (1.15–1.60) | 1.40 (1.16–1.69) | <0.001 |
|  | Never smoked | 1 | 1.14 (0.96–1.36) | 1.42 (1.19–1.69) | 1.46 (1.21–1.75) | 1.61 (1.30–1.99) | <0.001 | 1 | 1.20 (1.02–1.42) | 1.44 (1.21–1.70) | 1.35 (1.14–1.60) | 1.40 (1.15–1.70) | <0.001 |
| No. cancer in bladder | | 612/2,135 | 846/3,017 | 904/3,159 | 1,221/4,119 | 1,320/4,208 |  | 237/259 | 461/489 | 531/572 | 884/939 | 1,260/1,354 |  |
|  | Entire | 1 | 1.14 (1.05–1.23) | 1.17 (1.08–1.27) | 1.26 (1.16–1.37) | 1.38 (1.25–1.52) | <0.001 | 1 | 1.19 (0.97–1.45) | 1.20 (0.98–1.47) | 1.17 (0.96–1.43) | 1.27 (1.01–1.59) | 0.048 |
|  | Never smoked | 1 | 1.11 (0.96–1.28) | 1.15 (0.99–1.33) | 1.27 (1.09–1.48) | 1.45 (1.22–1.73) | <0.001 | 1 | 1.20 (0.98–1.48) | 1.19 (0.96–1.47) | 1.18 (0.95–1.45) | 1.27 (1.00–1.60) | 0.066 |
| No. leukemia | | 253/755 | 362/1,001 | 328/996 | 384/1,203 | 374/1,085 |  | 409/434 | 591/620 | 590/615 | 815/855 | 974/1,012 |  |
|  | Entire | 1 | 1.03 (0.90–1.17) | 1.04 (0.91–1.20) | 1.09 (0.94–1.26) | 1.11 (0.93–1.33) | 0.087 | 1 | 1.11 (0.94–1.31) | 1.12 (0.94–1.34) | 1.07 (0.89–1.29) | 1.06 (0.85–1.31) | 0.899 |
|  | Never smoked | 1 | 1.14 (0.92–1.43) | 1.07 (0.84–1.36) | 1.10 (0.85–1.42) | 1.23 (0.91–1.67) | 0.248 | 1 | 1.10 (0.93–1.31) | 1.11 (0.93–1.33) | 1.05 (0.87–1.26) | 1.04 (0.83–1.29) | 0.879 |
| No. cancer in cervix | |  |  |  |  |  |  | 2,206/2,459 | 2,740/2,986 | 2,493/2,693 | 3,356/3,587 | 3,805/4,117 |  |
|  | Entire | 1 | — |  |  |  |  | 1 | 1.05 (0.98–1.13) | 1.05 (0.97–1.14) | 1.05 (0.96–1.14) | 1.10 (1.00–1.22) | 0.051 |
|  | Never smoked | 1 | — |  |  |  |  | 1 | 1.05 (0.97–1.14) | 1.05 (0.97–1.14) | 1.05 (0.96–1.14) | 1.09 (0.99–1.21) | 0.089 |
| *Other cancers* | |  |  |  |  |  |  |  |  |  |  |  |  |
| No. malignant melanoma | | 61/153 | 80/230 | 108/311 | 130/356 | 129/353 |  | 168/181 | 195/207 | 243/253 | 396/410 | 493/509 |  |
|  | Entire | 1 | 1.15 (0.87–1.51) | 1.52 (1.15–2.01) | 1.45 (1.01–1.95) | 1.56 (1.10–2.21) | <0.001 | 1 | 0.78 (0.59–1.01) | 0.87 (0.67–1.15) | 0.90 (0.69–1.18) | 0.90 (0.66–1.23) | 0.880 |
|  | Never smoked | 1 | 1.02 (0.65–1.61) | 1.35 (0.86–2.13) | 1.36 (0.85–2.20) | 1.47 (0.84–2.57) | 0.030 | 1 | 0.78 (0.59–1.03) | 0.90 (0.68–1.19) | 0.94 (0.71–1.24) | 0.96 (0.69–1.32) | 0.496 |
| No. cancer in brain | | 241/783 | 324/1,014 | 304/996 | 370/1,215 | 371/1,173 |  | 558/600 | 805/855 | 890/930 | 1,282/1,345 | 1,632/1,706 |  |
|  | Entire | 1 | 1.06 (0.93–1.20) | 1.09 (0.95–1.25) | 1.18 (1.02–1.36) | 1.34 (1.12–1.60) | <0.001 | 1 | 1.10 (0.96–1.27) | 1.22 (1.05–1.14) | 1.21 (1.04–1.41) | 1.32 (1.11–1.58) | <0.001 |
|  | Never smoked | 1 | 1.08 (0.86–1.36) | 1.04 (0.82–1.34) | 1.11 (0.86–1.45) | 1.29 (0.94–1.76) | 0.068 | 1 | 1.10 (0.95–1.27) | 1.23 (1.06–1.43) | 1.22 (1.04–1.42) | 1.33 (1.11–1.60) | <0.001 |

“Entire” is the same value as in Table 2.

No. cancer presents as ‘cases in never smokers’/‘cases in entire population’.

**Table S2.** Influence of excluding cancer cases within the first 2 years during the follow-up periods

|  |  | Male |  |  |  |  |  | Female |  |  |  |  |  |
| --- | --- | --- | --- | --- | --- | --- | --- | --- | --- | --- | --- | --- | --- |
|  | | Q1 | Q2 | Q3 | Q4 | Q5 | *P*_trend_ | Q1 | Q2 | Q3 | Q4 | Q5 | *P*_trend_ |
| No. overall cancer | | 30,940/52,220 | 44,539/72,638 | 46,974/76,014 | 58,231/94,007 | 55,734/90,321 |  | 29,503/49,394 | 40,964/69,656 | 39,747/67,691 | 55,135/93,804 | 61,807/104,126 |  |
|  | Main | 1 | 1.10 (1.08–1.12) | 1.15 (1.14–1.17) | 1.21 (1.19–1.23) | 1.30 (1.27–1.33) | <0.001 | 1 | 1.15 (1.13–1.17) | 1.19 (1.17–1.21) | 1.17 (1.15–1.19) | 1.14 (1.12–1.17) | <0.001 |
|  | 2-yr exclusion | 1 | 1.11 (1.09–1.13) | 1.16 (1.14–1.19) | 1.22 (1.19–1.24) | 1.28 (1.25–1.32) | <0.001 | 1 | 1.13 (1.11–1.15) | 1.17 (1.14–1.19) | 1.16 (1.13–1.18) | 1.14 (1.11–1.17) | <0.001 |
| No. cancer in oral cavity | | 813/1,245 | 1,096/1,619 | 1,043/1,560 | 1,210/1,829 | 1,115/1,656 |  | 232/371 | 348/533 | 350/525 | 516/784 | 585/859 |  |
|  | Main | 1 | 1.13 (1.02–1.25) | 1.16 (1.04–1.30) | 1.23 (1.10–1.39) | 1.38 (1.20–1.59) | <0.001 | 1 | 1.10 (0.92–1.31) | 1.09 (0.90–1.32) | 1.12 (0.92–1.36) | 1.06 (0.84–1.33) | 0.624 |
|  | 2-yr exclusion | 1 | 1.15 (1.01–1.30) | 1.17 (1.02–1.34) | 1.22 (1.06–1.41) | 1.39 (1.17–1.65) | <0.001 | 1 | 1.15 (0.92–1.44) | 1.17 (0.93–1.49) | 1.20 (0.94–1.53) | 1.21 (0.91–1.60) | 0.142 |
| No. cancer in larynx | | 510/759 | 598/913 | 577/874 | 731/1,094 | 650/970 |  | 26/31 | 35/49 | 45/67 | 37/64 | 64/105 |  |
|  | Main | 1 | 1.11 (0.97–1.27) | 1.15 (1.00–1.33) | 1.31 (1.12–1.53) | 1.44 (1.20–1.73) | <0.001 | 1 | 1.15 (0.63–2.11) | 1.52 (0.83–2.78) | 0.96 (0.50–1.85) | 1.35 (0.65–2.79) | 0.665 |
|  | 2-yr exclusion | 1 | 1.05 (0.90–1.24) | 1.09 (0.91–1.30) | 1.24 (1.03–1.49) | 1.34 (1.07–1.68) | <0.001 | 1 | 0.95 (0.48–1.88) | 1.15 (0.57–2.30) | 0.61 (0.28–1.33) | 0.89 (0.38–2.11) | 0.332 |
| No. cancer in esophagus | | 867/1,522 | 1,046/1,773 | 933/1,484 | 1,078/1,713 | 914/1,475 |  | 55/92 | 87/132 | 73/128 | 125/190 | 152/237 |  |
|  | Main | 1 | 1.17 (1.07–1.29) | 1.12 (1.01–1.25) | 1.25 (1.11–1.40) | 1.47 (1.28–1.69) | <0.001 | 1 | 1.04 (0.73–1.49) | 0.98 (0.67–1.43) | 0.98 (0.66–1.43) | 1.10 (0.71–1.72) | 0.751 |
|  | 2-yr exclusion | 1 | 1.16 (1.02–1.31) | 1.15 (1.01–1.32) | 1.26 (1.08–1.46) | 1.41 (1.18–1.68) | <0.001 | 1 | 1.10 (0.70–1.73) | 0.87 (0.53–1.43) | 0.98 (0.60–1.60) | 1.03 (0.59–1.82) | 0.915 |
| No. cancer in stomach | | 7,240/13,544 | 9,939/17,782 | 10,433/18,213 | 12,613/21,898 | 11,412/20,122 |  | 2,216/4,069 | 3,535/6,402 | 3,697/6,685 | 5,937/10,584 | 7,543/13,294 |  |
|  | Main | 1 | 1.05 (1.02–1.08) | 1.07 (1.04–1.11) | 1.10 (1.06–1.14) | 1.12 (1.08–1.17) | <0.001 | 1 | 1.13 (1.07–1.19) | 1.13 (1.07–1.20) | 1.19 (1.12–1.26) | 1.26 (1.18–1.34) | <0.001 |
|  | 2-yr exclusion | 1 | 1.06 (1.02–1.11) | 1.10 (1.05–1.15) | 1.12 (1.07–1.17) | 1.11 (1.05–1.17) | <0.001 | 1 | 1.12 (1.04–1.20) | 1.12 (1.04–1.21) | 1.18 (1.10–1.27) | 1.25 (1.15–1.36) | <0.001 |
| No. cancer in colorectum | | 6,379/10,803 | 9,841/15,809 | 10,565/17,189 | 13,342/21,888 | 13,117/21,324 |  | 4,035/6,283 | 6,193/9,785 | 6,692/10,577 | 9,906/15,846 | 12,611/19,893 |  |
|  | Main | 1 | 1.14 (1.10–1.18) | 1.22 (1.18–1.27) | 1.31 (1.26–1.36) | 1.40 (1.34–1.50) | <0.001 | 1 | 1.12 (1.07–1.17) | 1.17 (1.12–1.22) | 1.15 (1.10–1.21) | 1.19 (1.13–1.26) | <0.001 |
|  | 2-yr exclusion | 1 | 1.17 (1.13–1.23) | 1.24 (1.18–1.29) | 1.30 (1.24–1.37) | 1.38 (1.31–1.46) | <0.001 | 1 | 1.09 (1.04–1.15) | 1.14 (1.08–1.21) | 1.11 (1.05–1.18) | 1.16 (1.09–1.24) | <0.001 |
| No. cancer in liver | | 3,721/5,857 | 5,334/8,458 | 5,599/8,659 | 6,810/10,524 | 7,107/10,939 |  | 923/1,410 | 1,617/2,378 | 1,852/2,731 | 3,006/4,484 | 4,288/6,233 |  |
|  | Main | 1 | 1.15 (1.10–1.20) | 1.18 (1.12–1.24) | 1.21 (1.15–1.28) | 1.38 (1.30–1.47) | <0.001 | 1 | 1.11 (1.02–1.22) | 1.16 (1.06–1.27) | 1.18 (1.08–1.29) | 1.22 (1.10–1.35) | <0.001 |
|  | 2-yr exclusion | 1 | 1.10 (1.04–1.16) | 1.14 (1.07–1.21) | 1.15 (1.08–1.23) | 1.28 (1.19–1.38) | <0.001 | 1 | 1.15 (1.03–1.28) | 1.19 (1.07–1.33) | 1.19 (1.07–1.33) | 1.25 (1.10–1.41) | <0.001 |
| No. cancer in biliary tract | | 860/1,354 | 1,291/1,945 | 1,471/2,218 | 1,959/2,870 | 1,896/2,768 |  | 393/584 | 748/1,085 | 940/1,392 | 1,668/2,489 | 2,334/3,476 |  |
|  | Main | 1 | 1.12 (1.02–1.23) | 1.22 (1.11–1.35) | 1.29 (1.16–1.42) | 1.28 (1.14–1.45) | <0.001 | 1 | 1.12 (0.98–1.28) | 1.22 (1.07–1.39) | 1.27 (1.11–1.45) | 1.25 (1.08–1.45) | <0.001 |
|  | 2-yr exclusion | 1 | 1.13 (1.00–1.27) | 1.22 (1.08–1.37) | 1.30 (1.15–1.47) | 1.27 (1.10–1.48) | <0.001 | 1 | 1.14 (0.97–1.34) | 1.21 (1.03–1.43) | 1.26 (1.07–1.48) | 1.25 (1.04–1.49) | 0.003 |
| No. cancer in pancreas | | 2,446/3,712 | 3,470/5,150 | 3,816/5,573 | 4,769/6,820 | 4,487/6,381 |  | 1,092/1,649 | 1,912/2,759 | 2,054/2,958 | 3,370/4,798 | 4,266/6,127 |  |
|  | Main | 1 | 1.08 (1.02–1.15) | 1.16 (1.09–1.23) | 1.19 (1.11–1.27) | 1.21 (1.12–1.31) | <0.001 | 1 | 1.14 (1.05–1.24) | 1.13 (1.04–1.23) | 1.16 (1.06–1.26) | 1.16 (1.05–1.27) | 0.004 |
|  | 2-yr exclusion | 1 | 1.08 (1.00–1.16) | 1.16 (1.08–1.25) | 1.20 (1.11–1.30) | 1.21 (1.10–1.33) | <0.001 | 1 | 1.17 (1.06–1.30) | 1.16 (1.05–1.29) | 1.20 (1.08–1.33) | 1.18 (1.05–1.32) | 0.009 |
| No. cancer in lung | | 5,291/8,160 | 6,414/9,501 | 6,363/9,453 | 7,872/11,471 | 7,133/10,413 |  | 1,512/2,173 | 2,491/3,622 | 2,660/3,894 | 4,130/6,129 | 5,072/7,317 |  |
|  | Main | 1 | 1.09 (1.05–1.13) | 1.17 (1.12–1.22) | 1.29 (1.23–1.35) | 1.44 (1.36–1.52) | <0.001 | 1 | 1.15 (1.07–1.23) | 1.16 (1.07–1.24) | 1.17 (1.09–1.26) | 1.16 (1.06–1.26) | 0.001 |
|  | 2-yr exclusion | 1 | 1.10 (1.05–1.16) | 1.17 (1.11–1.23) | 1.29 (1.22–1.37) | 1.42 (1.33–1.52) | <0.001 | 1 | 1.14 (1.04–1.24) | 1.14 (1.04–1.25) | 1.14 (1.05–1.25) | 1.16 (1.05–1.29) | 0.006 |
| No. cancer in kidney | | 674/1,102 | 1,265/1,990 | 1,550/2,344 | 2,033/3,110 | 2,288/3,450 |  | 272/417 | 445/698 | 581/889 | 903/1,339 | 1,243/1,865 |  |
|  | Main | 1 | 1.27 (1.15–1.40) | 1.41 (1.28–1.57) | 1.50 (1.35–1.67) | 1.66 (1.47–1.88) | <0.001 | 1 | 1.19 (1.01–1.40) | 1.42 (1.21–1.68) | 1.36 (1.15–1.60) | 1.40 (1.16–1.69) | <0.001 |
|  | 2-yr exclusion | 1 | 1.30 (1.14–1.47) | 1.50 (1.32–1.70) | 1.57 91.37–1.79) | 1.74 (1.50–2.03) | <0.001 | 1 | 1.12 (0.92–1.37) | 1.35 (1.11–1.66) | 1.31 (1.07–1.60) | 1.28 (1.01–1.61) | 0.007 |
| No. cancer in bladder | | 1,360/2,135 | 2,016/3,017 | 2,138/3,159 | 2,797/4,119 | 2,900/4,208 |  | 165/259 | 335/489 | 398/572 | 613/939 | 928/1,354 |  |
|  | Main | 1 | 1.14 (1.05–1.23) | 1.17 (1.08–1.27) | 1.26 (1.16–1.37) | 1.38 (1.25–1.52) | <0.001 | 1 | 1.19 (0.97–1.45) | 1.20 (0.98–1.47) | 1.17 (0.96–1.43) | 1.27 (1.01–1.59) | 0.048 |
|  | 2-yr exclusion | 1 | 1.17 (1.06–1.28) | 1.20 (1.09–1.33) | 1.29 (1.17–1.43) | 1.41 (1.25–1.59) | <0.001 | 1 | 1.27 (0.99–1.62) | 1.30 (1.01–1.66) | 1.19 (0.93–1.53) | 1.35 (1.02–1.78) | 0.087 |
| No. cancer in thyroid | | 2,204/3,499 | 3,826/6,097 | 4,046/6,525 | 5,004/8,096 | 4,863/7,967 |  | 10,943/19,022 | 14,245/25,509 | 12,784/23,095 | 16,092/29,019 | 15,276/27,486 |  |
|  | Main | 1 | 1.33 (1.25–1.40) | 1.49 (1.40–1.58) | 1.60 (1.50–1.70) | 1.68 (1.56–1.81) | <0.001 | 1 | 1.27 (1.23–1.30) | 1.36 (1.32–1.39) | 1.33 (1.29–1.37) | 1.16 (1.12–1.21) | <0.001 |
|  | 2-yr exclusion | 1 | 1.32 (1.223–1.42) | 1.47 (1.36–1.58) | 1.58 (1.46–1.71) | 1.64 (1.49–1.81) | <0.001 | 1 | 1.24 (1.20–1.29) | 1.33 (1.28–1.38) | 1.32 (1.27–1.38) | 1.17 (1.12–1.23) | <0.001 |
| No. cancer in brain | | 506/783 | 667/1,014 | 673/996 | 802/1,215 | 789/1,173 |  | 387/600 | 568/855 | 631/930 | 888/1,345 | 1,159/1,706 |  |
|  | Main | 1 | 1.06 (0.93–1.20) | 1.09 (0.95–1.25) | 1.18 (1.02–1.36) | 1.34 (1.12–1.60) | <0.001 | 1 | 1.10 (0.96–1.27) | 1.22 (1.05–1.14) | 1.21 (1.04–1.41) | 1.32 (1.11–1.58) | <0.001 |
|  | 2-yr exclusion | 1 | 1.06 (0.90–1.24) | 1.11 (0.94–1.32) | 1.16 (0.97–1.39) | 1.33 (1.07–1.66) | <0.001 | 1 | 1.12 (0.94–1.33) | 1.25 (1.05–1.50) | 1.21 (1.00–1.45) | 1.34 (1.08–1.66) | 0.001 |
| No. malignant melanoma | | 103/153 | 159/230 | 220/311 | 246/356 | 238/353 |  | 115/181 | 152/207 | 180/253 | 268/410 | 356/509 |  |
|  | Main | 1 | 1.15 (0.87–1.51) | 1.52 (1.15–2.01) | 1.45 (1.01–1.95) | 1.56 (1.10–2.21) | <0.001 | 1 | 0.78 (0.59–1.01) | 0.87 (0.67–1.15) | 0.90 (0.69–1.18) | 0.90 (0.66–1.23) | 0.880 |
|  | 2-yr exclusion | 1 | 1.20 (0.86–1.68) | 1.66 (1.18–2.33) | 1.58 (1.10–2.27) | 1.72 (1.13–2.62) | <0.001 | 1 | 0.90 (0.65–1.24) | 0.98 (0.70–1.37) | 0.93 (0.67–1.31) | 1.02 (0.69–1.50) | 0.714 |
| No. lymphoma | | 782/1,213 | 1,112/1,648 | 1,133/1,716 | 1,446/2,147 | 1,294/1,995 |  | 426/690 | 748/1,178 | 689/1,134 | 1,097/1,686 | 1,241/1,918 |  |
|  | Main | 1 | 1.05 (0.95–1.16) | 1.10 (0.99–1.23) | 1.18 (1.05–1.32) | 1.23 (1.07–1.41) | <0.001 | 1 | 1.31 (1.16–1.49) | 1.30 (1.13–1.48) | 1.34 (1.17–1.54) | 1.35 (1.15–1.58) | <0.001 |
|  | 2-yr exclusion | 1 | 1.08 (0.95–1.22) | 1.10 (0.97–1.26) | 1.20 (1.04–1.38) | 1.20 (1.01–1.42) | 0.002 | 1 | 1.33 (1.13–1.56) | 1.24 (1.04–1.47) | 1.36 (1.14–1.61) | 1.34 (1.09–1.64) | 0.005 |
| No. leukemia | | 468/755 | 676/1,001 | 688/996 | 848/1,203 | 752/1,085 |  | 286/434 | 387/620 | 418/615 | 572/855 | 722/1,012 |  |
|  | Main | 1 | 1.03 (0.90–1.17) | 1.04 (0.91–1.20) | 1.09 (0.94–1.26) | 1.11 (0.93–1.33) | 0.087 | 1 | 1.11 (0.94–1.31) | 1.12 (0.94–1.34) | 1.07 (0.89–1.29) | 1.06 (0.85–1.31) | 0.899 |
|  | 2-yr exclusion | 1 | 1.10 (0.93–1.29) | 1.13 (0.95–1.31) | 1.19 (0.99–1.43) | 1.17 (0.94–1.46) | 0.042 | 1 | 1.00 (0.82–1.24) | 1.08 (0.87–1.34) | 0.99 (0.79–1.24) | 1.00 (0.77–1.30) | 0.833 |
| No. multiple myeloma | | 268/405 | 376/517 | 410/576 | 509/707 | 495/679 |  | 123/176 | 206/304 | 284/403 | 472/653 | 594/796 |  |
|  | Main | 1 | 0.95 (0.80–1.14) | 1.01 (0.84–1.22) | 1.01 (0.83–1.23) | 1.00 (0.79–1.26) | 0.701 | 1 | 1.14 (0.89–1.47) | 1.38 (1.08–1.77) | 1.40 (1.10–1.80) | 1.36 (1.03–1.80) | 0.003 |
|  | 2-yr exclusion | 1 | 1.02 (0.82–1.26) | 1.05 (0.84–1.31) | 1.05 (0.83–1.32) | 1.03 (0.78–1.37) | 0.712 | 1 | 1.10 (0.81–1.48) | 1.37 (1.02–1.84) | 1.43 (1.06–1.92) | 1.43 (1.02–1.99) | 0.002 |
| No. cancer in prostate | | 4,429/6,531 | 6,577/9,295 | 7,296/10,375 | 9,455/13,359 | 9,080/12,932 |  | — |  |  |  |  |  |
|  | Main | 1 | 1.09 (1.05–1.14) | 1.16 (1.10–1.21) | 1.19 (1.14–1.25) | 1.17 (1.11–1.23) | <0.001 |  |  |  |  |  |  |
|  | 2-yr exclusion | 1 | 1.12 (1.07–1.18) | 1.17 (1.11–1.24) | 1.21 (1.15–1.28) | 1.18 (1.10–1.26) | <0.001 |  |  |  |  |  |  |
| No. cancer in testis | | 159/227 | 235/293 | 230/298 | 287/346 | 273/341 |  | — |  |  |  |  |  |
|  | Main | 1 | 1.00 (0.79–1.27) | 1.05 (0.82–1.36) | 1.06 (0.81–1.40) | 1.20 (0.86–1.67) | 0.149 |  |  |  |  |  |  |
|  | 2-yr exclusion | 1 | 1.10 (0.84–1.45) | 1.08 (0.80–1.45) | 1.14 (0.83–1.55) | 1.19 (0.82–1.73) | 0.281 |  |  |  |  |  |  |
| No. preMP cancer in breast | | — |  |  |  |  |  | 5,761/8,897 | 6,342/9,958 | 4,480/7,083 | 4,204/6,775 | 2,766/4,479 |  |
|  | Main |  |  |  |  |  |  | 1 | 1.05 (1.01–1.09) | 1.06 (1.02–1.11) | 1.07 (1.02–1.13) | 1.05 (0.98–1.13) | 0.007 |
|  | 2-yr exclusion |  |  |  |  |  |  | 1 | 1.06 (1.01–1.11) | 1.08 (1.02–1.14) | 1.07 (1.00–1.14) | 1.06 (0.97–1.16) | 0.026 |
| No. postMP cancer in breast | | — |  |  |  |  |  | 1,674/2,942 | 3,225/5,674 | 3,953/6,778 | 6,020/10,365 | 6,974/11,884 |  |
|  | Main |  |  |  |  |  |  | 1 | 0.95 (0.89–1.01) | 0.96 (0.90–1.02) | 0.95 (0.89–1.00) | 0.93 (0.87–0.99) | 0.035 |
|  | 2-yr exclusion |  |  |  |  |  |  | 1 | 0.94 (0.87–1.01) | 0.97 (0.90–1.05) | 0.95 (0.88–1.03) | 0.95 (0.87–1.04) | 0.518 |
| No. preMP cancer in uterus | | — |  |  |  |  |  | 487/692 | 547/795 | 399/580 | 474/684 | 532/824 |  |
|  | Main |  |  |  |  |  |  | 1 | 0.95 (0.83–1.09) | 0.87 (0.74–1.02) | 0.93 (0.78–1.09) | 1.13 (0.92–1.39) | 0.546 |
|  | 2-yr exclusion |  |  |  |  |  |  | 1 | 0.93 (0.79–1.10) | 0.86 (0.71–1.04) | 0.93 (0.76–1.13) | 1.08 (0.84–1.38) | 0.833 |
| No. postMP cancer in uterus | | — |  |  |  |  |  | 235/408 | 515/834 | 608/1,024 | 1,103/1,612 | 1,443/2,273 |  |
|  | Main |  |  |  |  |  |  | 1 | 0.96 (0.82–1.12) | 0.95 (0.82–1.12) | 0.93 (0.79–1.08) | 1.02 (0.86–1.21) | 0.695 |
|  | 2-yr exclusion |  |  |  |  |  |  | 1 | 1.02 (0.83–1.25) | 0.97 (0.79–1.19) | 1.00 (0.81–1.22) | 1.10 (0.88–1.38) | 0.212 |
| No. preMP cancer in ovary | | — |  |  |  |  |  | 866/1,269 | 888/1,261 | 589/886 | 611/882 | 554/799 |  |
|  | Main |  |  |  |  |  |  | 1 | 0.97 (0.87–1.08) | 0.97 (0.85–1.10) | 0.99 (0.86–1.14) | 1.23 (1.03–1.47) | 0.072 |
|  | 2-yr exclusion |  |  |  |  |  |  | 1 | 1.00 (0.88–1.13) | 0.94 (0.81–1.09) | 1.00 (0.77–1.08) | 1.24 (1.00–1.53) | 0.169 |
| No. postMP cancer in ovary | | — |  |  |  |  |  | 375/597 | 707/1,102 | 958/1,447 | 1,371/2,164 | 1,792/2,748 |  |
|  | Main |  |  |  |  |  |  | 1 | 0.93 (0.81–1.06) | 1.02 (0.89–1.16) | 0.96 (0.84–1.09) | 1.03 (0.89–1.20) | 0.299 |
|  | 2-yr exclusion |  |  |  |  |  |  | 1 | 0.92 (0.78–1.09) | 1.03 (0.87–1.21) | 0.91 (0.77–1.08) | 0.99 (0.82–1.19) | 0.918 |
| No. cancer in cervix | | — |  |  |  |  |  | 1,433/2,459 | 1,681/2,986 | 1,469/2,693 | 1,927/3,587 | 2,248/4,117 |  |
|  | Main |  |  |  |  |  |  | 1 | 1.05 (0.98–1.13) | 1.05 (0.97–1.14) | 1.05 (0.96–1.14) | 1.10 (1.00–1.22) | 0.051 |
|  | 2-yr exclusion |  |  |  |  |  |  | 1 | 1.05 (0.95–1.15) | 1.04 (0.93–1.16) | 1.04 (0.93–1.16) | 1.15 (1.01–1.32) | 0.034 |

MP, menopausal.

“Main” is the same value in Table 2.

No. cancer presents as ‘cases in 2-yr exclusion’/‘cases in main’.
